# Between-groups within-gene heterogeneity of residual variances in microarray gene expression data

**DOI:** 10.1186/1471-2164-9-319

**Published:** 2008-07-04

**Authors:** Joaquim Casellas, Luis Varona

**Affiliations:** 1 Genètica i Millora Animal, IRTA-Lleida, 25198 Lleida, Spain; 2 Departamento de Anatomía, Embriología y Genética, Universidad de Zaragoza, 50013 Zaragoza, Spain

## Abstract

**Background:**

The analysis of microarray gene expression data typically tries to identify differential gene expression patterns in terms of differences of the mathematical expectation between groups of arrays (e.g. treatments or biological conditions). Nevertheless, the differential expression pattern could also be characterized by group-specific dispersion patterns, although little is known about this phenomenon in microarray data. Commonly, a homogeneous gene-specific residual variance is assumed in hierarchical mixed models for gene expression data, although it could result in substantial biases if this assumption is not true.

**Results:**

In this manuscript, a hierarchical mixed model with within-gene heterogeneous residual variances is proposed to analyze gene expression data from non-competitive hybridized microarrays. Moreover, a straightforward Bayes factor is adapted to easily check within-gene (between groups) heterogeneity of residual variances when samples are grouped in two different treatments. This Bayes factor only requires the analysis of the complex model (hierarchical mixed model with between-groups heterogeneous residual variances for all analyzed genes) and gene-specific Bayes factors are provided from the output of a simple Markov chain Monte Carlo sampling.

**Conclusion:**

This statistical development opens new research possibilities within the gene expression framework, where heterogeneity in residual variability could be viewed as an alternative and plausible characterization of differential expression patterns.

## Background

Gene expression measured by microarray chips is an emerging and cost-effective tool to assess the expression of thousands of genes in different tissues and organisms [1]. This technology has been intensively used to monitor changes in gene expression between tissues, treatments or time points in order to detect genes, or even metabolic pathways, involved in differential expression patterns [2]. As was highlighted by Wolfinger et al. [3], inference in microarray gene expression analyses is typically focused on gene-specific differences between mathematical expectations of two (or more) groups of biological conditions. However, discrepancies in gene expression could also be characterized by other statistics of interest like dispersion parameters [4,5].

Heterogeneity of residual variances is a topic of main concern in biological studies where residual variance changes under alternative treatments [6,7]. In gene expression analyses, heterogeneity of gene-specific residual dispersion has been addressed recently [8,9], where hierarchical mixed models with gene-specific residual variances substantially reduced the rate of false positives and allowed for a more realistic fit of gene expression data [10]. Nevertheless, a common within-gene residual variance was assumed in these analyses, although within-gene discrepancies in the dispersion parameters could also be feasible. To our best knowledge, discrepancies in terms of gene-specific residual variance across different biological conditions (groups or arrays) have never been considered in the microarray literature. Besides a plausible scale effect on the residual variance due to changes in mathematical expectation under different groups of microarrays, within-gene heterogeneity of the residual variance could suggest a group-specific pattern of variability at the transcription level. Variability could be just due to within tissue variability in cell type composition, but may or may not be related to any meaningful difference in transcription.

The aim of this research is to propose a hierarchical mixed model analysis of microarray gene expression data assuming within-gene heterogeneous residual variances. In addition, a straightforward Bayes factor approach to test differences between two within-gene residual variances is developed, taking Verdinelli and Wasserman [11] and Varona et al. [12] as starting point. This methodology could open a new research field in gene expression analysis where differential gene expression will be characterized in terms of variability of the transcription process.

## Methods

### Hierarchical mixed model with within-gene heterogeneous residual variances

Assume as starting point *n *replicates of non-competitive hybridization microarray data with *m *genes (or probes; each probe is a fragment of complementary nucleic acid covering genomic or inter-genomic annotated regions) per array. Under the simplest design, these replicates are distributed in two different groups of treatments (e.g. tissues, species or time points) with *r *and *s *replicates per treatment, respectively (*r *+ *s *= *n*). This gene expression data can be analyzed under the following hierarchical mixed model [13],

**y **= **Xa **+ **Z**_1_**p**_1 _+ **Z**_2_**p**_2 _**+e**,

where **y **is the (*nm*) × 1 column vector of intensity scores sorted by array within treatment within gene and **e **is the (*nm*) × 1 column vector of residuals. Effects in model were array (**a**; dimension *n *× 1) and probe (**p**_1 _and **p**_2_; dimension *m *× 1) linked to **y **by appropriate incidence matrices (**X**, **Z**_1 _and **Z**_2_, respectively). Vector **e **is assumed to be normally distributed [14],

**e **~ *N*(0, **R**),

**R **being the matrix of residual (co)variances. Assuming null residual (co)variances [8,9,13] and heterogeneous gene-specific residual variances between treatments, **R **can be stated as

$$R=\overset{m}{\underset{i=1}{⊕}}\left[\begin{array}{cc} I_{1}\sigma_{e\left(i1\right)}^{2} & 0\\ 0^{\prime } & I_{2}\sigma_{e\left(i2\right)}^{2} \end{array}\right],$$

where **I**_1 _is a *r *× *r *identity matrix, **I**_2 _is a *s *× *s *identity matrix, **0 **is a *r *× *s *matrix of zeros, and $\sigma_{e\left(ij\right)}^{2}$ is the residual variance for the *i*th gene and *j*th treatment. Under a standard Bayesian development, the joint posterior probability of all unknowns in model is proportional to

$$\begin{array}{c} p\left(a,p_{1},p_{2},R,\sigma_{p1}^{2},\sigma_{p2}^{2},|y\right)\propto p\left(y|a,p,d\left(p\right),R\right)p\left(a\right)p\left(p_{1}|\sigma_{p1}^{2}\right)p\left(\sigma_{p1}^{2}\right)\\ \times p\left(p_{2}|\sigma_{p2}^{2}\right)p\left(\sigma_{p2}^{2}\right)p\left(R\right) \end{array},$$

with a flat prior for **a **and multivariate normal *a priori *distributions for **y**, **p**_1 _and **p**_2 _[13],

$$\begin{array}{l} p\left(y|a,p_{1},p_{2},R\right)\sim N(Xa+Z_{1}p_{1}+Z_{2}p_{2},R),\\ p\left(p_{1}|\sigma_{p1}^{2}\right)\sim N\left(0,I_{m}\sigma_{p1}^{2}\right), \end{array}$$

and

$$p\left(p_{2}|\sigma_{p2}^{2}\right)\sim N\left(0,I_{m}\sigma_{p2}^{2}\right),$$

where **I**_*m *_is an *m *× *m *identity matrix, and $\sigma_{p1}^{2}$ and $\sigma_{p2}^{2}$ are the variance components for **p**_1 _and **p**_2_, respectively. Additionally, inverted *χ*^2 ^priors with hyperparameters *S*^2 ^and *ν *are assumed for variance components,

$$\begin{array}{l} p\left(\sigma_{p1}^{2}\right)\sim \chi_{S_{p1}^{2},\nu_{p1}}^{-2},\\ p\left(\sigma_{p2}^{2}\right)\sim \chi_{S_{p2}^{2},\nu_{p2}}^{-2}, \end{array}$$

and

$$p\left(R\right)\sim \prod_{i=1}^{m}\prod_{j=1}^{2}\chi_{S_{e\left(ij\right)}^{2},\nu_{e\left(ij\right)}}^{-2}.$$

All the unknowns in model can be sampled under a Markov chain Monte Carlo framework by standard Gibbs sampling [15].

### Bayes factor to test within-gene heterogeneous residual variances

When gene expression data is grouped in two different treatments or groups the Verdinelli and Wasserman's [11] approach to Bayes factor can be easily adapted. In order to allow for a straightforward comparison between $\sigma_{e\left(i1\right)}^{2}=\sigma_{e\left(i2\right)}^{2}$ and $\sigma_{e\left(i1\right)}^{2}\neq \sigma_{e\left(i2\right)}^{2}$ hypothesis and without loss of generality, $R=\overset{m}{\underset{i=1}{⊕}}\left[\begin{array}{cc} I_{1}\sigma_{e\left(i1\right)}^{2} & 0\\ 0^{\prime } & I_{2}\sigma_{e\left(i2\right)}^{2} \end{array}\right]$ can be redefined as

$$R^{*}=\overset{m}{\underset{i=1}{⊕}}\left[\begin{array}{cc} I_{1}\sigma_{e\left(i\right)}^{2}\pi_{i} & 0\\ 0^{\prime } & I_{2}\sigma_{e\left(i\right)}^{2}\left(1-\pi_{i}\right) \end{array}\right],$$

and consequently, $\sigma_{e\left(i\right)}^{2}=\sigma_{e\left(i1\right)}^{2}+\sigma_{e\left(i2\right)}^{2}$ and $\pi_{i}=\sigma_{e\left(i1\right)}^{2}/\sigma_{e\left(i\right)}^{2}$. Note *π*_*i *_can be viewed as a variance heterogeneity parameter where *π*_*i *_= 0.5 accounts for equal residual variances between treatments and *π*_*i *_≠ 0.5 suggests within-gene (between treatments) heterogeneity of residual variances. Assuming $\sigma^{\prime }=\left[\begin{array}{cccc} \sigma_{e\left(1\right)}^{2} & \sigma_{e\left(2\right)}^{2} & \ldots & \sigma_{e\left(m\right)}^{2} \end{array}\right]$ and **σ' **= [*π*_1 _*π*_2 _... *π*_*m*_], this reparameterization can also be developed within a Bayesian frame, with the following joint posterior probability,

$$\begin{array}{c} p\left(a,p_{1},p_{2},\sigma ,\pi ,\sigma_{p1}^{2},\sigma_{p2}^{2},|y\right)\propto p\left(y|a,p_{1},p_{2},R^{*}\right)p\left(a\right)p\left(p_{1}|\sigma_{p1}^{2}\right)p\left(\sigma_{p1}^{2}\right)\\ \times p\left(p_{2}|\sigma_{p2}^{2}\right)p\left(\sigma_{p2}^{2}\right)p\left(\sigma \right)p\left(\pi \right) \end{array},$$

and Bayesian likelihood,

*p*(**y**|**a**,**p**_1_,**p**_2_,R*) ~ *N*(**Xa **+ **Z**_1_**p**_1 _+ **Z**_2_**p**_2_, R*).

We assume the same prior distributions for **a**, **p**_1_, **p**_2_, $\sigma_{p1}^{2}$ and $\sigma_{p2}^{2}$ as in previous model, and a scaled inverted *χ*^2 ^prior for elements in **σ**

$$p\left(\sigma \right)\sim \prod_{i=1}^{m}\chi_{S_{e\left(i\right)}^{2},\nu_{e\left(i\right)}}^{-2}.$$

Note that this parameterization allows for a gene-specific definition of hyperparameters $S_{e\left(i\right)}^{2}$ and *ν*_*e*(*i*)_, modifying the shape of the inverted scaled *χ*^2 ^prior accordingly to our a priori knowledge about the dispersion patter of each gene. Nevertheless, if we lack of a priori information about gene-specific dispersion patterns, this prior could be reduced to a proper flat distribution with appropriate bound. The priori distribution for **π **is stated as flat between appropriate bounds,

$$\begin{array}{cc} p\left(\pi \right)\sim \prod_{i=1}^{m}1 & \text{if }\pi_{i}\in \left[0,\;1\right]\text{ and 0 otherwise}\text{.} \end{array}$$

Note that this prior distribution is the key point for the further calculation of the Bayes factor and covers all possible values taken by *π*_*i *_with equal probability, following Verdinelli and Wasserman [11] and Varona et al. [12]. As in previous model parameterization, all unknowns can be updated by Gibbs sampling [15] with the exception of *π*_*i *_that requires a Metropolis-Hastings step [16].

For a given gene, model comparison between $\sigma_{e\left(i1\right)}^{2}\neq \sigma_{e\left(i2\right)}^{2}$ and $\sigma_{e\left(i1\right)}^{2}=\sigma_{e\left(i2\right)}^{2}$ hypotheses simplifies to conditions *π*_*i *_≠ 0.5 (within-gene heterogeneous residual variances for all genes; Model HE) and *π*_*i *_= 0.5 (homogeneous residual variance for the *i*th gene, within-gene heterogeneous residual variances for the remaining genes; Model HO_*i*_). Note that *π*_*i *_is assumed known and fixed in Model HO_*i *_and then, Model HO_*i *_and Model HE are nested models that only differ in a bounded variable (*π*_*i*_). It is important to highlight that this Bayes factor testes gene-by-gene dispersion patterns, although it does not inform us about the best analytical model for the joint inference of all genes. Following the methodology developed by Verdinelli and Wasserman [11], the Bayes factor between Model HE and Model HO_*i *_(${\text{BF}}_{{\text{HE/HO}}_{i}}$) can be easily calculated from the Markov chain Monte Carlo sampler output of Model HE, by averaging the full conditional densities of each cycle at *π*_*i *_= 0.5 using the Rao-Blackwell argument [17]. Following García-Cortés et al. [18] and Varona et al. [12], the posterior density *p*(*π*_*i *_= 0.5|**y**) suffices to obtain ${\text{BF}}_{{\text{HE/HO}}_{i}}$,

$${\text{BF}}_{{\text{HE/HO}}_{i}}=\frac{p\left(\pi_{i}=0.5\right)}{p\left(\pi_{i}=0.5|y\right)}=\frac{1}{p\left(\pi_{i}=0.5|y\right)},$$

because *p*(*π*_*i *_= 0.5) was previously defined with the *a priori *distribution of *π*_*i*_. On the basis of GEAMM v.1.4 program [13], the Bayes factor developed above was implemented with FORTRAN90 language. All the subsequent analyses were performed with this software.

### Example 1. Simulated data

Our Bayes factor approach was tested on simulated data sets under three different scenarios in order to check its statistical performance. For each scenario, a total of 100 data sets were generated, each one including 40 arrays (unrelated individuals), 10,000 genes per array and two groups of treatments (A and B). More specifically, scenario 1 (S1) assigned 20 arrays to each treatment without missing data, scenario 2 (S2) assumed an unbalanced design with 10 and 30 arrays for treatments A and B, respectively (no missing data), and scenario 3 (S3) assumed two groups of 20 arrays with a 5% of randomly distributed missing data. Intensity scores (*y*_*ijk*_) were simulated under the following model,

*y*_*ijk *_= *μ *+ *a*_*i *_+ *g*_*j *_= *e*_*ijk*_,

were *μ *was the overall mean arbitrarily fitted to 6, *a*_*i *_was the effect of each array sampled from an uniform distribution between 0 and 1, *g*_*j *_was the effect of the gene sampled from a Gaussian distribution with mean 0 and variance 1, and *e*_*ijk *_was the residual term obtained from a Gaussian distribution with mean zero and variance 0.1 × *π*_*i *_(Group A) or 0.1 × (1 - *π*_*i*_) (Group B). We assumed a unique (and plausible) value for the overall residual variance in order to allow for a direct comparison and interpretations of the results. Genes were grouped in five levels with different values of *π*_*i*_: 0.5 (6,000 genes), 0.4 (1,000 genes), 0.3 (1,000 genes), 0.2 (1,000 genes) and 0.1 (1,000 genes). Statistical performance of the developed BF to check differential expression in terms of dispersion pattern (residual variances) was compared with a well-known standard *F*-test. The effect of within-gene differential expression was not considered in order to allow for a straightforward comparison between Group A and Group B residual variances under *F*-test. Indeed, additional sources of variation were avoided in order to allow for a direct calculation of the *F*-test without requiring preliminary pre-correction of the data. Each data set was analyzed by the Bayes factor approach described above with the scaled *χ*^-2 ^prior distribution for variances components generalized to proper flat priors (*S*^2 ^= 0 and *ν *= -2) defined between > 0 and 1000. A unique Monte Carlo Markov chain with 100,000 elements was launched for each data set, after discarding the first 10,000 iterations as burn-in. Convergence was checked by the Raftery and Lewis [19] algorithm.

### Example 2. Free-access gene expression data

To illustrate the methodology described above, we applied the model to free-access fibroblast gene expression data from 10 chimps and 11 gorillas (available at Gene Expression Omnibus [20], accession number GDS340). As described Karaman et al. [21], hybridization was performed in the Human Genome U95 Set platform (Affymetrix, Santa Clara, CA). A rough normalization was performed on the original data set by calculating multiplicative scaling factors on the basis of the median intensity of the 60th and 95th quintile of gene-expression scores [21]. All gene-expression scores below 100 were set to 100 in the original data set (untransformed scale) and. Within this context, all genes with one or more scores equal to 100 were removed from the final analysis. After editing, data set included gene expression scores of 3,700 genes, transformed by a base-2-logarithm as suggested Yeung et al. [22]. The analytical process followed the same specifications as for simulated data sets.

## Results

### Example 1. Simulated data

As can be seen in Table 1, differences between simulated and predicted values of π were small, suggesting a reasonable model adjustment to gene expression data. Indeed, the average posterior mean for the residual variance was 0.102 (the empirical standard error across-genes and replicates was 0.002; S1) and agreed with the value used in simulations (0.1). When gene expression data was generated under equal residual variances across groups (*π *= 0.5), the Bayes factor (${\text{BF}}_{{\text{HE/HO}}_{i}}$) discarded heterogeneous variances in the greater part of the cases (S1: 88% to 98%; S2: 90% to 98%; S3: 90% to 97%). Under S1 and following Jeffreys' [23] scale of evidence, between 1% and 12% of genes reached vague evidences of heterogeneous variances and only between 1% and 3% of genes showed substantial evidences of heterogeneous variances (Table 1). Results under S2 (unbalanced design) and S3 (missing data) provided a similar trend with and expectable power loss (Table 1). Although a small percentage of genes supported the existence of heterogeneous variances, these results do not invalidate our Bayes factor approach, given that a substantial increase in false positives is expected when the number of replicates (arrays) per analyses is small, a typical phenomenon in microarray data sets. Moreover, these results agreed with the ones obtained by a standard *F*-test, where a 1–12% of genes (across data sets and simulation scenarios) reached *p*-values lower than 0.05.

**Table 1:** Simulated (*π*) and predicted ($\tilde{\pi }$; average of the posterior mean across-genes and replicates) heterogeneity and percentage of genes falling within each category of the Bayes Factor for the three simulation scenarios

			1 ≥	3.16 ≥	10 ≥	31.62 ≥	
*π*	$\tilde{\pi }$^1^	${\text{BF}}_{{\text{HE/HO}}_{i}}$	${\text{BF}}_{{\text{HE/HO}}_{i}}$	${\text{BF}}_{{\text{HE/HO}}_{i}}$	${\text{BF}}_{{\text{HE/HO}}_{i}}$	${\text{BF}}_{{\text{HE/HO}}_{i}}$	${\text{BF}}_{{\text{HE/HO}}_{i}}$
		< 1	< 3.16	< 10	< 31.62	< 100	≥ 100
Simulation scenario 1							
0.5	0.498	88 to 98	1 to 12	1 to 3	0	0	0
0.4	0.412	60 to 84	2 to 28	1 to 16	0 to 8	0 to 4	0 to 4
0.3	0.301	20 to 48	16 to 36	12 to 28	4 to 12	0 to 12	0 to 16
0.2	0.196	4 to 16	8 to 32	4 to 28	4 to 24	8 to 28	16 to 36
0.1	0.121	0	0 to 4	0 to 4	0 to 8	0 to 8	84 to 100

Simulation scenario 2							
0.5	0.492	90 to 98	2 to 11	0 to 2	0 to 1	0	0
0.4	0.407	64 to 89	1 to 22	0 to 11	0 to 7	0 to 2	0 to 1
0.3	0.296	23 to 51	11 to 27	9 to 22	2 to 9	0 to 8	0 to 7
0.2	0.201	10 to 21	7 to 30	2 to 25	2 to 25	3 to 27	9 to 31
0.1	0.115	0 to 1	0 to 8	0 to 10	0 to 12	0 to 17	76 to 100

Simulation scenario 3							
0.5	0.508	90 to 97	1 to 11	1 to 4	0	0	0
0.4	0.409	62 to 87	1 to 26	0 to 14	0 to 6	0 to 4	0 to 3
0.3	0.308	23 to 51	15 to 34	10 to 25	3 to 10	0 to 9	0 to 10
0.2	0.211	5 to 17	7 to 31	4 to 26	4 to 23	7 to 26	14 to 31
0.1	0.119	0 to 1	0 to 8	0 to 8	0 to 10	0 to 11	77 to 100

^1^Empirical standard errors were smaller than 0.01 in all cases.

As was expected, ${\text{BF}}_{{\text{HE/HO}}_{i}}$ showed an overall increase when *π *values used in the simulation process decreased (Table 1). The percentage of ${\text{BF}}_{{\text{HE/HO}}_{i}}$ < 1 decreased with *π*, it ranging between 60% and 84% (*π *= 0.4), between 20% and 48% (*π *= 0.3), between 4% and 16% (*π *= 0.2) and 0% (*π *= 0.1). Additionally, evidences favoring the presence of heterogeneous variances increased when gene expression data were simulated under small *π*, 84% to 100% of the genes reaching ${\text{BF}}_{{\text{HE/HO}}_{i}}$ ≥ 100 for *π *= 0.1 simulated genes (decisive evidence according to Jeffreys's [23] scale). This increase in ${\text{BF}}_{{\text{HE/HO}}_{i}}$ when the bounded variable (*π*) departed from the "null hypothesis" (*π *= 0.5) agrees with previous results published by García-Cortés et al. [18] and Casellas et al. [24] with the same Bayes factor approach although adapted to test heritability of linear and threshold traits.

**Figure 1:**
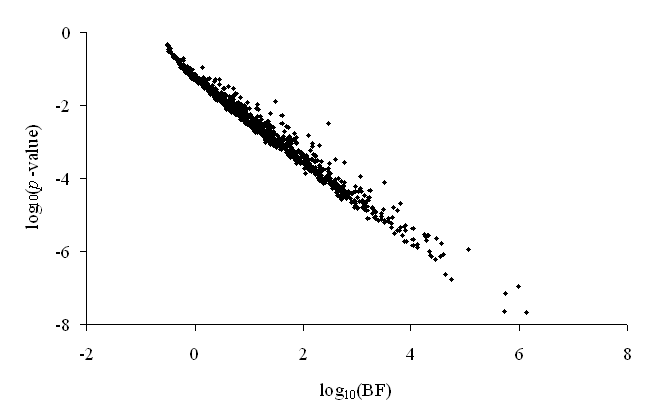
Plot of log_10_(*p*-value) against log_10_(BF) for residual variance comparison in the first simulated data set of S1.

As can be seen in Figure 1, our Bayes factor and the standard *F*-test performed similarly, in contrast to the noticeable computational instability of previous approximations to the Bayes factor [25]. Nevertheless, the approximation adapted in this manuscript has been previously compared with other statistics of reference like likelihood ratio test [12] or the deviance information criterion [13] developed by Spiegelhalter et al. [26], and showed a very similar performance without detecting remarkable deviations. The strong similarity between the proposed Bayes factor and the standard *F*-test could the viewed as a critical advantage for the *F*-test under a very simple microarray design with two different treatments. When additional sources of variation are included in model, the proposed Bayes factor takes advantage of the joint analysis for all the parameters in the model and simultaneous testing for discrepancies between residual variances of interest. Within this scenario, the *F*-test requires a previous pre-correction for additional sources of variation in the model and therefore, implies a two-steps analysis.

### Example 2. Free-access gene expression data

**Figure 2:**
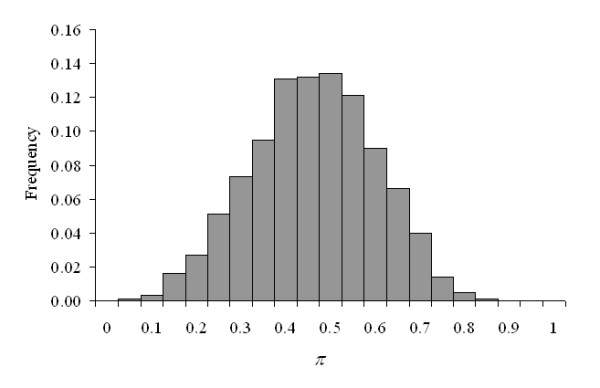
Distribution of *π *values for gene expression analysis of fibroblast data between chimps and gorillas.

**Figure 3:**
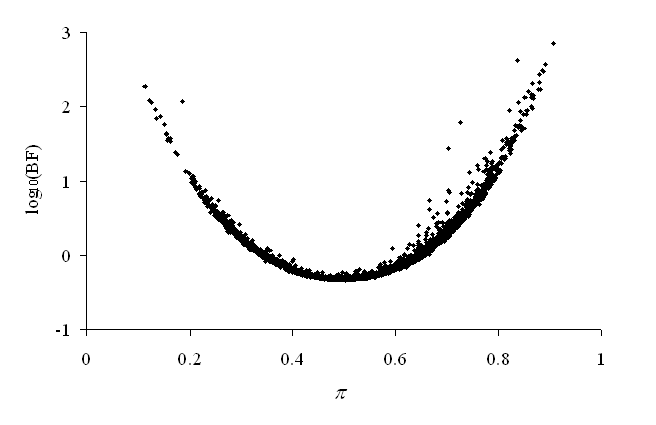
Plot of log_10_(BF) against *π *(posterior mean) for the analysis of gene expression data between chimps and gorillas.

Results are shown in Table 2, where 67.9% of genes did not reveal evidence of within-gene heterogeneous residual variances, and 20.1% of genes suggested almost discernable deviations from Model HO_*i *_(1 ≥ ${\text{BF}}_{{\text{HE/HO}}_{i}}$ < 3.16). It is graphically illustrated in Figure 2 where most of the estimated *π *values were accumulated around 0.5, the value characterizing within-gene homogeneous residual variances. Nevertheless, substantial (8.2% of genes), strong (2.2%), very strong (1.1%) and decisive evidences (0.6%) of within-gene heterogeneous residual variances following Jeffreys' [23] scale were detected (Table 2; Figure 3) in this free-access data set.

**Table 2:** Distribution of genes according to BF_HE/Ho*i*_, and across-genes average estimates (and empirical standard error across average estimates) for the heterogeneity parameter and residual variance

${\text{BF}}_{{\text{HE/HO}}_{i}}$	Genes	π*^1^	$\sigma_{e\left(i\right)}^{2}$
${\text{BF}}_{{\text{HE/HO}}_{i}}$ < 1	2,511^2 ^(67.9)^3^	0.431 (0.001)	0.244 (0.005)
1 ≥ ${\text{BF}}_{{\text{HE/HO}}_{i}}$ < 3.16	743 (20.1)	0.316 (0.001)	0.243 (0.008)
3.16 ≥ ${\text{BF}}_{{\text{HE/HO}}_{i}}$ < 10	302 (8.2)	0.249 (0.001)	0.241 (0.013)
10 ≥ ${\text{BF}}_{{\text{HE/HO}}_{i}}$ < 31.62	80 (2.2)	0.262 (0.022)	0.725 (0.021)
31.62 ≥ ${\text{BF}}_{{\text{HE/HO}}_{i}}$ < 100	41 (1.1)	0.275 (0.030)	0.665 (0.050)
${\text{BF}}_{{\text{HE/HO}}_{i}}$ ≥ 100	23 (0.6)	0.278 (0.042)	0.744 (0.061)

^1^Values were transformed to 1 - *π *when *π *was grater than 0.5.

^2^Number of genes.

^3^Percentage related to the overall number of genes analyzed.

As was expected, the across-genes average *π *values (transformed to 1 - *π *when *π *was greater than 0.5) was maximum for genes with ${\text{BF}}_{{\text{HE/HO}}_{i}}$ < 1 (0.431), whereas this parameter reduced to around 0.25 when ${\text{BF}}_{{\text{HE/HO}}_{i}}$ increased (Table 2). Note that in our analyses, extreme values of *π *values (< 0.1 or > 0.9) were scarce (Figure 2). For the joint residual variance ($\sigma_{e\left(i\right)}^{2}$), averages ranged between 0.241 and 0.744 (Table 2).

## Discussion

### Within-gene heterogeneity of the residual dispersion patter in real data

Results obtained in the comparison between chimp and gorilla gene expression data suggested a substantial incidence of within-gene heterogeneity, which is not typically accounted for in standard gene expression analyses. Moreover, detection of relevant (or significant) genes was substantially affected by the analytical model, as is illustrated in Table 3. Model HE showed a more conservative pattern and, when ${\text{BF}}_{{\text{HE/HO}}_{i}}$ took greater-than-one values, this phenomenon suggested that the rate of false positives increased if within-gene heterogeneity of residual variances was not accounted for in the model [10,27]. A moderate percentage of genes with heterogeneous residual variances did not show differences in terms of mathematical expectation (Table 3), therefore discarding a scale effect. Although these results can not be directly extrapolated to all microarray data sets, these results suggests that heterogeneous residual patterns could be a biological phenomenon of special interest in further analysis of gene expression data. Variability could be just due to within tissue variability in cell type composition, but may or may not be related to any meaningful difference in transcription.

**Table 3:** Distribution of relevant genes according to BF_HE/Ho*i *_and under two different analytical models

	Posterior probability^1 ^< 0.05		Posterior probability^1 ^< 10^-5^	
${\text{BF}}_{{\text{HE/HO}}_{i}}$	Model HE	Model HO^2^	Model HE	Model HO^2^
${\text{BF}}_{{\text{HE/HO}}_{i}}$ < 1	995	1058	210	240
1 ≥ ${\text{BF}}_{{\text{HE/HO}}_{i}}$ < 3.16	337	349	73	79
3.16 ≥ ${\text{BF}}_{{\text{HE/HO}}_{i}}$ < 10	161	168	31	33
10 ≥ ${\text{BF}}_{{\text{HE/HO}}_{i}}$ < 31.62	45	46	10	10
31.62 ≥ ${\text{BF}}_{{\text{HE/HO}}_{i}}$ < 100	28	32	2	4
${\text{BF}}_{{\text{HE/HO}}_{i}}$ ≥ 100	8	9	3	4

^1^Posterior probability above (greater-than-zero average difference) of below zero (lower-than-zero average difference) of the difference between chip *vs*. gorilla mathematical expectation.

^2^All *π*_*i *_were arbitrarily fitted to 0.5.

### Bayes factor to compare dispersion patterns in microarray studies

Although gene expression analyses have been typically focused on the comparison between mathematical expectations of two or more (within-gene) groups of arrays, the analytical approach developed in the present paper allow for an alternative characterization of differential expression patterns. Moreover, it allows for an appropriate data modeling when within-gene heterogeneity of residual variances exists. This approach could be viewed as statistically inefficient for those genes with homogeneity of residual variances [28,29]. Nevertheless, the aim of this research was to provide an accurate method to compare dispersion patterns, whereas differences between the mathematical expectances of groups of treatments are not of interest in this case. This test could also be applied to experiments with less replicates per group although its results must be taken with caution, given the inherent loss of robustness under small data sets,. As is shown in Figure 1, our Bayes factor performed similarly to the standard *F*-test, with a stable a coherent behavior under moderate sample sizes (number or arrays per group). Although the Bayes factor approach has been described under a simple scenario (simulated datasets), this can be easily generalized to more complex frameworks without additional requirements. Within this context, across-genes shrinkage of residual variances is a topic of main interest in microarray research [29,30] which can be easily adapted to the hierarchical mixed model applied above. Indeed, several Bayesian methods proposed for residual variances shrinkage [31,32] can be applied to both residual variances and heterogeneity parameters, and the calculation of the Bayes factor does not substantially change within-gene or within a group of genes. In a similar way, other approaches can also be jointly implemented with the developed Bayes factor such as mixtures of distributions [33-35]. If several sources of variation are expected on the residual term, the mixed model with within-gene heterogeneous residual variances could be viewed as a useful tool to characterize their distribution pattern, the Bayes factor being a straightforward way to check their statistical relevance. Within this context, our Bayes factor procedure could provide preliminary results required for the application of more complex and computational demanding approaches like the mixed model with log-transformed hierarchical residual variances developed by Jaffrezic et al. [36].

Changes in residual dispersion patterns could be due to a scale effect when mathematical expectations of two (or more) groups of arrays are different. Nevertheless, this scale-related hypothesis was only attributable to a small percentage of genes with heterogeneous residual variances (Tables 3), whereas more than 75% of differential dispersion patters must be related to other unknown causes in the analyzed free-access microarray data. These changes in the dispersion pattern were previously suggested in genes involved in cancer pathogenesis [4,37] and other diseases [38], although within-gene residual heterogeneity is not commonly considered in gene expression analyses [8,9]. Moreover, heterogeneity in gene expression increases with age [5] and therefore, our approach could be of special interest in time-series analyses where individuals at different ages are compared. As a whole, the hierarchical mixed model with within-gene heterogeneous residual variances allows for a new and more accurate modeling of gene expression data with appealing perspectives, and the Bayes factor developed is an easy way to check differences between within-gene residual variances.

Under the Bayesian framework, model comparison is usually made by calculating Bayes factors [39], the ratio between the marginal probabilities of the data given the tested models and after integrating out all parameters in the models. The Bayes factor developed by Verdinelli and Wasserman [11] from generalization of the Savage-Dickey density ratio, and adapted to the animal breeding context by García-Cortés et al. [18] and Varona et al. [12], has been easily applied to check heterogeneous residual variances in gene expression analyses when two groups of treatments are compared. It provides a rigorous and clear framework to compare competing models, avoiding the calculation of significance levels and without depending upon asymptotic properties of frequentist estimators [40], Bayes factor behaves well even when the bounded variable to be tested is either close to the boundary of the parametric space [18]. In addition, Bayes factor provides a ratio of probabilities between models, without any requirement to define the null or the alternative hypothesis, without trying to discard the null hypothesis in favor of an alternative hypothesis, and without referring to the asymptotic properties of the frequentist estimators [12].

Although other Bayes factor approaches could be used, the Verdinelli and Wasserman's [11] approach allows for a simplified calculation, where only the analysis of the complex model is necessary. Moreover, a unique analysis is required to calculate all the gene-specific Bayes factors, and chain storage is not needed because only the (within-gene) average of the full conditional densities at *π*_*i *_= 0.5 is used during calculation. Under alternative Bayes factor approaches [39], an additional model with *π *= 0.5 for the gene to be tested (and sampling *π *for the remaining genes) must be analyzed for each gene, in order to obtain the gene-specific Bayes factor comparing heterogeneous *versus *homogeneous residual variances.

Given the null a priori information about the expected distribution of **π**, we assumed a flat prior distribution between 0 and 1 in order to give the same a priori probability to all plausible values. This is a standard assumption for the Verdinelli and Wasserman's Bayes factor [11,12], although other prior distributions could also become reasonable. It is important to note that *p*(**π**) equally influences both *p*(*π*_*i *_= 0.5) and p(*π*_*i *_= 0.5|**y**) terms and therefore, the Bayes factor must be relatively robust to prior modifications. In the light of the results obtained from the analysis of great ape gene expression data, a priori distributions favoring values close to 0.5 and with decreasing probability in their tails could be realistic. Within this context, Gaussian, Laplace and Student's *t *distributions with mean 0.5 and truncated at 0 and 1 could be useful a priori distributions, among others. Nevertheless, further studies are required to confirm this pattern in real gene expression data.

As was recently demonstrated at the gene-specific level [10], an accurate modeling of residual dispersion allows for a more realistic fit of gene expression data. Moreover, it has a relevant impact on the rate of false positives when gene expression is characterized in terms of mathematical expectations or their differences [8-10]. In this manuscript, we have adapted Lo and Gottardo [10] mixed model to account for within gene heterogeneity of residual variances, where a relevant incidence of within-gene heterogeneity has been revealed in real gene expression data. Moreover, this heterogeneity can be easily checked gene-by-gene by applying a straightforward Bayes factor with a minimal increase in computational requirements. Note that differences between average gene expression without assuming equal residual variances is a typical example of the Behrens-Fisher problem [41], which could be easily by-passed in microarray analyses by appropriately adapting Welch's [42]*t-*test. Nevertheless, our approach seeks a novel point of view were, not only differences between mathematical means are tested but differences between residual dispersion patterns must also be checked and characterized. In addition, our Bayes factor allows to detect those genes with heterogeneous residual variances where Behrens-Fisher problem [41] holds.

## Conclusion

Accounting for within-gene between-groups heterogeneous residual variances in mixed model analyses of non-competitive microarray gene expression data (or even competitive microarray gene expression data after suitable data editing) allows to characterize differential expression patterns in terms of gene expression variability. The Bayes factor approach here presented provides a straightforward comparison between within-gene group-specific residual variances with minimal computing requirements. This methodology is freely available in GEAMM v.1.7 software [43].

## Authors' contributions

The methodological approach described in this manuscript was developed by both JC and LV. In addition, JC implemented the analytical model and simulated data sets in FORTRAN90 language, evaluated both real and simulated data sets and drafted the manuscript. LV and contributed to the interpretation of the analyses results and manuscript preparation. Both authors read and approved the final manuscript.
